# Demonstration of the Protective Effect of Vinpocetine in Diabetic Cardiomyopathy

**DOI:** 10.3390/jcm13164637

**Published:** 2024-08-08

**Authors:** Demet Erciyes, Ejder Saylav Bora, Mustafa Agah Tekindal, Oytun Erbaş

**Affiliations:** 1 Department of Cardiology, Faculty of Medicine, Demiroğlu Bilim University, 34394 Istanbul, Türkiye; demet.erciyes@demioglu.bilim.edu.tr; 2 Department of Emergency Medicine, Faculty of Medicine, Izmir Katip Çelebi University, 35620 Izmir, Türkiye; 3 Department of Basic Medical Sciences Biostatistics, Faculty of Medicine, İzmir Katip Çelebi Unıversity, 35620 Izmir, Türkiye; mustafaagah.tekidal@ikc.edu.tr; 4 Department of Physiology, Faculty of Medicine, Demiroğlu Bilim University, 34394 Istanbul, Türkiye; oytunerbas2012@gmail.com

**Keywords:** diabetic cardiomyopathy, vinpocetine, TGF-β, cardioprotective

## Abstract

**Background**: Diabetic cardiomyopathy (DCM) poses a significant risk for heart failure in individuals with diabetes, yet its underlying mechanisms remain incompletely understood. Elevated blood sugar levels initiate harmful processes, including apoptosis, collagen accumulation, and fibrosis in the heart. Vinpocetine, a derivative of *Vinca minor* L., has demonstrated diverse pharmacological effects, including vasodilation, anti-inflammatory properties, and enhanced cellular metabolism. This study aims to investigate Vinpocetine’s protective and remodeling effects in diabetic cardiomyopathy by evaluating biochemical and histopathological parameters. **Methods**: Twenty-one adult male Wistar rats were induced with diabetes using streptozocin and divided into Diabetes and Diabetes + Vinpocetine groups. Histopathological analyses, TGF-β1 immunoexpression, and measurements of plasma markers (TGF-β, pro-BNP, Troponin T) were performed. Biochemical analyses included HIF-1 alpha and neuregulin-1β quantification and evaluation of lipid peroxidation. **Results**: Vinpocetine significantly reduced cardiac muscle thickness, TGF-β1 expression, and plasma in diabetic rats. HIF-1 alpha and neuregulin-1β levels increased with Vinpocetine treatment. Histopathological observations confirmed reduced fibrosis and structural abnormalities in Vinpocetine-treated hearts. **Conclusions**: This study provides comprehensive evidence supporting the protective effects of Vinpocetine against diabetic cardiomyopathy. Vinpocetine treatment improved cardiac morphology, immunohistochemistry, and modulation of biochemical markers, suggesting its potential as a therapeutic intervention to attenuate the negative impact of diabetes on heart function.

## 1. Introduction

The incidence of heart failure in diabetes is increasing day by day and is known to be between 20 and 26% [1]. Diabetic cardiomyopathy (DCM), a condition that occurs in individuals with diabetes without the presence of coronary artery disease or hypertension, is a significant contributor to heart failure [2]. Although this complication is significant, the underlying mechanisms are not well comprehended. Mounting evidence indicates that elevated blood sugar levels play a key role in the development of diabetic cardiomyopathy. Consequently, it is suggested that incorporating the dysfunctional contractile function of the diabetic cardiomyocyte into the definition of DCM would enhance its comprehensiveness and conclusiveness [3,4]. This initiates a cascade of harmful processes, including programmed cell death (apoptosis) of heart muscle cells, accumulation of collagen, and the formation of scar tissue (fibrosis) in the heart [4,5]. 

The presence of myocardial fibrosis is the defining characteristic of diabetic cardiomyopathy. Several symptoms are associated with this condition, including the accumulation of rigid collagen with cross-linking, the development of cardiac interstitial fibrosis, the disappearance of muscular fibrils, the presence of fibrosis around blood vessels, the thickening and hardening of small coronary vessels, the thickening of the basement membrane, and the occurrence of coronary microvascular sclerosis and microaneurysms [6,7].

During the beginning stages of diabetic cardiomyopathy, it is not uncommon for patients to not display any symptoms that are not bothersome. One of the initial signs is a decrease in the flexibility of the left ventricle, which is characterized by an enlargement of the left ventricle. Other signs include a delay in filling during the early phase of diastole, an increase in filling in the atrium, and a prolonged period of relaxation without any volume change [8]. The development of left ventricular dilatation and symptomatic heart failure are both preceded by the presence of systolic dysfunction [8].

Vinpocetine-ethyl apovicaminate was first created by Lorincz and his colleagues by synthesizing the alkaloid Vinpocetine, derived from the leaves of *Vinca minor* L. [9]. Vinpocetine has multiple pharmacological effects, such as increasing blood flow in the brain as a vasodilator, enhancing cerebral metabolism by promoting glucose and oxygen absorption, and stimulating ATP synthesis in neurons [10,11,12,13]. Furthermore, it functions as a potent anti-inflammatory substance in different types of cells, including epithelial cells, vascular smooth muscle cells, endothelial cells, and macrophages [13]. Previous studies have shown that Vinpocetine causes the widening of blood vessels and prevents abnormal tissue growth in the blood vessels and the hardening of arteries in rodents in the outer parts of the circulatory system [14,15,16]. 

NRG1, known as Neuregulin-1, is a potent stimulator of cardiomyocyte proliferation. It is produced and released by endothelial vascular cells, significantly affecting the cardiovascular system. It significantly influences heart development, cell division, specialization, programmed cell death, and other biological processes related to the cardiovascular system [17]. Hypoxia-inducible factor 1 (HIF-1) is a transcription factor that centrally controls oxygen balance in all multicellular organisms. HIF-1 governs the transportation of oxygen by overseeing the growth of new blood vessels (angiogenesis) and the restructuring of existing blood vessels (vascular remodeling).

Additionally, HIF-1 regulates oxygen utilization by controlling glucose metabolism and maintaining a balance in the redox state [18]. Expression of TGF-β in the myocardium is increased in experimental models of myocardial infarction (MI) and cardiac hypertrophy, as well as in patients with dilated or hypertrophic cardiomyopathy. TGF-β significantly impacts cardiomyocytes, mesenchymal cells, and immune cells, and it plays a crucial role in developing cardiac remodeling and fibrosis. The overexpression of TGF-β in the mouse heart is linked to the development of fibrosis and hypertrophy [19].

In the present investigation, our objective is to demonstrate Vinpocetine’s protective and remodeling effect in diabetic cardiomyopathy. We will accomplish this by analyzing biochemical and histopathologic parameters.

## 2. Materials and Methods

### 2.1. Animals

The study utilized 21 adult male Wistar rats with a weight range of 200–210 g. The animals were confined in cages and kept under controlled conditions with 12 h periods of light and darkness at a temperature of 22 ± 2 °C. The subjects were provided with a standard pellet diet and had unrestricted access to tap water throughout the study. The study protocol was approved by the Institutional Animal Care and Ethical Committee of the University of Science University (Ethical Number: 2823100922). The chemicals were acquired from Sigma-Aldrich Inc. (Saint Louis, MO, USA) unless specified otherwise.

### 2.2. Experimental Protocol

When 14 rats were given an injection of streptozocin (STZ, Sigma-Aldrich, Inc.; Saint Louis, MO, USA) intraperitoneally (i.p.) at a dose of 60 mg/kg in a solution of 0.9% sodium chloride, the pH of the solution was adjusted to 4.0 with 0.2 M sodium citrate. This caused the rats to develop diabetes. The normal control group consisted of the seven rats left over from the study for which no medication was administered. Within twenty-four hours, the diagnosis of diabetes was confirmed by determining the glucose levels in the blood through the utilization of glucose oxidase reagent strips manufactured by Boehringer-Mannheim in Indianapolis. Within this study’s scope, rats whose blood glucose levels were equal to or higher than 250 mg/dL were included. Subsequently, a total of fourteen rats with diabetes were randomly assigned to two groups: the diabetes group, which received a saline solution at a dosage of 1 mL/kg (Diabetes) (n = 7), and the diabetes group, which received a daily dosage of 5 mg/kg of Vinpocetine (Diabetes + Vinpocetine) (n = 7), administered intraperitoneally for four weeks after initial administration. Upon completion of the study, blood samples from rats were obtained through tail vein puncture for biochemical analysis. Subsequently, all animals were euthanized using cervical dislocation as a sacrificial method. Anesthesia was induced using Ketamine (100 mg/kg, Ketasol, Richterpharma AG, Wels, Austria) and xylazine (50 mg/kg, Rompun, Bayer, Leverkusen, Germany). The hearts were then extracted for histopathological analysis. 

### 2.3. Histopathological Examination of Heart Tissue

First of all, after the heart was extracted, we cut the heart transversely into two slices through the center of the heart. Then, we took a 3 mm transverse slice from the base of the heart and measured the wall thickness (endocardium, myocardium, and epicardium) of the left and right ventricles and septum muscles. The left ventricular wall thickness of the normal group was accepted as 100 percent.

The remaining pieces of the heart were quickly immersed in a solution of 10% formaldehyde in 0.1 M phosphate-buffered saline (PBS) for three days. Heart sections (5 μm) preserved in formalin were stained using hematoxylin and eosin (H&E). The Olympus C-5050 digital camera (Shinjuku-ku, Tokyo, Japan) photographed all sections, mounted on the Olympus BX51 microscope (Shinjuku-ku, Tokyo, Japan). 

A computerized image analysis system was utilized to evaluate the morphological analysis. Through the use of light microscopy, the degree of hypertrophy that was present in cardiac myocytes was determined. Through the use of the cross-sectional image, the thickness of the muscle cells was accurately determined. Although the examiner was unaware of the origin of the tissue, he or she could obtain a picture of the cross-section that produced the largest diameter of the muscle fiber. A digital format was subsequently applied to the image after conversion. Image-Pro Express 1.4.5, an image analysis program developed in the United States by Media Cybernetics, Inc. (Rockville, MD, USA), was utilized to determine the number of muscle fibers. An average of fifty cardiac muscle cells were used for the analysis, which was carried out on each animal.

### 2.4. TGF-β1 Immunoexpression

During the immunohistochemistry procedure, the sections were subjected to a 10% solution of H_2_O_2_ for 30 min in order to eliminate the inherent peroxidase activity. Subsequently, they encountered an impediment in the form of a 10% solution of normal goat serum (Invitrogen) for 1 h at room temperature. Subsequently, the sections were immersed in primary antibodies (TGF-β1, Bioss, Inc., Woburn, MA, USA; 1/100) and kept at a temperature of 4 °C for 24 h. The Histostain-Plus Bulk kit from Bioss, Inc. detected antibodies targeting rabbit IgG. 3,3′ diaminobenzidine (DAB) was employed to visualize the resulting product. 

The sections were purified using phosphate-buffered saline (PBS) and recorded using an Olympus C-5050 digital camera connected to an Olympus BX51 microscope. Brown cytoplasmic staining revealed positive immunoexpression. 

The quantification of cells expressing positive immunoreactivity was conducted by systematically assessing at least fifty cardiac muscle cells per field in ten fields of tissue sections, utilizing a 40× magnification.

### 2.5. Measurement of Plasma TGF-β, Pro-BNP, Troponin T

TGF-β, pro-BNP, and Troponin T plasma levels were quantified using a commercially available enzyme-linked immunosorbent assay (ELISA) kit from Biosciences.

### 2.6. Heart Biochemical Analysis 

Following decapitation, the organs were promptly extracted and kept at a temperature of 20 °C until biochemical analyses were conducted. In order to analyze the tissue, the heart was crushed using a glass homogenizer in a solution of phosphate-buffered saline (PBS) that was five times the volume of the tissue. The pH of the solution was 7.4. The resulting mixture was spun at a centrifugal force of 5000× *g* for 15 min. The liquid portion was gathered, and the overall protein concentration within nerve homogenates was assessed using Bradford’s technique, with bovine serum albumin serving as the reference standard [20]. HIF-1 alpha and neuregulin-1β concentrations in the heart tissue supernatants were quantified using commercially available rat ELISA kits.

### 2.7. Evaluation of Lipid Peroxidation

Plasma samples were assessed for lipid peroxidation by quantifying malondialdehyde (MDA) levels as thiobarbituric acid reactive substance (TBARS). In summary, trichloroacetic acid and TBARS reagent were introduced to the plasma samples, followed by thorough mixing and incubation at 100 °C for 60 min. After cooling on ice, the samples underwent centrifugation at a speed of 3000 revolutions per minute for 20 min. The absorbance of the liquid portion above the sediment was then measured at a wavelength of 535 nanometers. The MDA levels were quantified in nanomolar units, with tetraethoxypropane as the calibration standard.

### 2.8. Statistical Analysis

The results of tests were expressed as the number of observations (n), mean ± standard deviation, median, and min-max values. The results of the homogeneity (Levene’s Test) and normality tests (Shapiro–Wilk) were used to decide which statistical methods to apply in the comparison of the study groups. Normally distributed and with homogeneous variances, groups were compared to three or more groups by Analysis of Variance. According to those test results, parametric test assumptions were unavailable for some variables, and the Kruskal–Wallis test performed comparisons of three independent groups. Multiple comparison tests, such as the adjusted Bonferroni test, were used. SPPS 25 (IBM Corp. Released 2017. IBM SPSS Statistics for Windows, Version 25.0. IBM Corp.: Armonk, NY, USA) statistical package program was used to evaluate the data. The significance level of the tests was assumed to be *p* < 0.05. 

## 3. Results

### 3.1. Cardiac Morphology

The morphological analysis was conducted on the Normal Control (Group-1), Diabetes and saline treatment (Group-2), and Diabetes and Vinpocetine (5 mg/kg) treatment (Group-3) groups (Table 1).

#### 3.1.1. Cardiac Muscle Cell Thickness

Cardiac muscle cell thickness, as a percentage of control and in micrometers, demonstrated significant alterations among the groups (*p* < 0.01). The Diabetes and saline treatment group exhibited notable increases compared to the Normal Control group, indicating hypertrophic changes (*p* < 0.01). Conversely, treatment with Vinpocetine in the Diabetes and Vinpocetine group effectively mitigated this hypertrophy, showing significantly reduced cardiac muscle cell thickness compared to both the Diabetes and saline treatment group and the Normal Control group (*p* < 0.01) (Table 1).

#### 3.1.2. Left Ventricular Thickness

Left ventricular thickness differed significantly among the groups (*p* < 0.01). Similar to cardiac muscle cell thickness, the Diabetes and saline treatment group demonstrated increased left ventricular thickness compared to the Normal Control group (*p* < 0.01). Notably, treatment with Vinpocetine in the Diabetes and Vinpocetine group attenuated this increase, resulting in thickness levels comparable to the Normal Control group (*p* < 0.01) (Table 1).

#### 3.1.3. Right Ventricular Thickness

Right ventricular thickness significantly varied among the groups (*p* < 0.01). The Diabetes and saline treatment group exhibited elevated thickness compared to the Normal Control group (*p* < 0.01). However, treatment with Vinpocetine in the Diabetes and Vinpocetine group effectively reduced right ventricular thickness, approaching levels observed in the Normal Control group (*p* < 0.01) (Table 1).

#### 3.1.4. Interventricular Septum Thickness

Interventricular septum thickness significantly differed among the groups (*p* < 0.01). Like other cardiac parameters, the Diabetes and saline treatment group exhibited increased septum thickness compared to the Normal Control group (*p* < 0.01). Treatment with Vinpocetine in the Diabetes and Vinpocetine group attenuated this increase, resulting in thickness levels similar to the Normal Control group (*p* < 0.01) (Table 1).

#### 3.1.5. Immunoexpression of TGF-β1

The immunoexpression of TGF-β1 exhibited a significant difference among the groups (*p* < 0.001). The Diabetes and saline treatment group showed a substantial increase in immunoexpression compared to the Normal Control group (*p* < 0.001). Conversely, treatment with Vinpocetine in the Diabetes and Vinpocetine group significantly attenuated TGF-β1 immunoexpression compared to both the Diabetes and saline treatment group and the Normal Control group (*p* < 0.001) (Table 2).

#### 3.1.6. Blood Glucose Levels

Blood glucose levels significantly differed among the groups (*p* < 0.01). The Diabetes and saline treatment group displayed markedly elevated blood glucose levels compared to the Normal Control group (*p* < 0.01). Conversely, treatment with Vinpocetine in the Diabetes and Vinpocetine group resulted in a significant reduction in blood glucose levels compared to the Diabetes and saline treatment group (*p* < 0.01) (Table 2).

### 3.2. Plasma Biomarkers

Plasma TGF-Beta levels significantly altered among the groups (*p* < 0.001). The Diabetes and saline treatment group exhibited substantially higher levels than the Normal Control group (*p* < 0.001). Treatment with Vinpocetine in the Diabetes and Vinpocetine group led to a significant reduction in TGF-Beta levels compared to both the Diabetes and saline treatment group and the Normal Control group (*p* < 0.001) (Table 2).

Plasma MDA levels demonstrated significant differences among the groups (*p* < 0.001). The Diabetes and saline treatment group displayed markedly elevated MDA levels compared to the Normal Control group (*p* < 0.001). Treatment with Vinpocetine in the Diabetes and Vinpocetine group resulted in a significant reduction in MDA levels compared to both the Diabetes and saline treatment group and the Normal Control group (*p* < 0.001) (Table 2).

### 3.3. Cardiac Biomarkers

Cardiac HIF-1alpha levels differed significantly among the groups (*p* < 0.01). Notably, the Diabetes and saline treatment group significantly reduced HIF-1alpha levels compared to the Normal Control group (*p* < 0.01). Conversely, treatment with Vinpocetine in the Diabetes and Vinpocetine group led to a significant increase in HIF-1alpha levels compared to the Diabetes and saline treatment group (*p* < 0.01), although remaining lower than the Normal Control group.

Cardiac neuregulin-1β levels were significantly altered among the groups (*p* < 0.01). The Diabetes and saline treatment group displayed a marked decrease compared to the Normal Control group (*p* < 0.01). Treatment with Vinpocetine in the Diabetes and Vinpocetine group showed a trend towards normalization of neuregulin-1β levels, although not reaching significance compared to the Diabetes and saline treatment group.

### 3.4. Microscopic Observations

**Figure 1:**
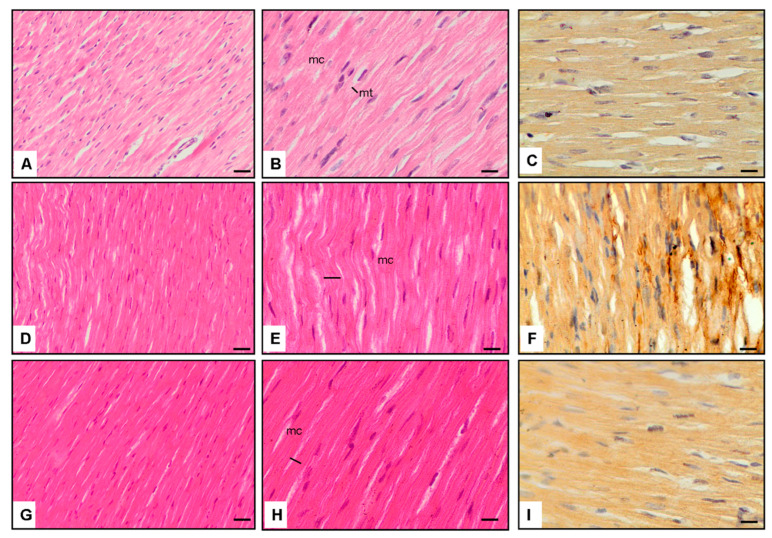
Cardiac tissue histopathology *Hematoxylin* and *Eosin stain* (×20 and ×40 magnification, 100 uM) and *TGF-β1* immunoexpression (Brown staining) in cardiomyocytes (×40 magnification). (**A**–**C**): Normal cardiomyocytes (mc) and thickness (mt), (**D**–**F**): Diabetic rats have increased thickness and *TGF-β1* expression of cardiomyocytes, (**G**–**I**): Vinpocetine-treated diabetic rats have decreased thickness and *TGF-β1* expression of cardiomyocytes (scale bar = 200 μm).

Panels A–C display normal cardiomyocytes (mc) and thickness (mt) in the Normal Control group (Group-1) at magnifications of ×20 and ×40. These sections reveal baseline cardiac morphology with regular cell size and minimal fibrotic changes (Figure 1).

Panels D–F exhibit cardiomyocytes from the Diabetes and saline group (Group-2) at ×40 magnification. In diabetic rats, an evident increase in cardiomyocyte thickness and TGF-β1 immunoexpression is observed, indicative of pathological alterations associated with diabetic cardiomyopathy (Figure 1).

Panels G–H–I showcase the cardiomyocytes of Vinpocetine-treated diabetic rats (Diabetes + Vinpocetine group, Group-3) at ×40 magnification. In this group, a notable reduction in cardiomyocyte thickness and TGF-β1 immunoexpression is apparent, suggesting a protective effect of Vinpocetine against diabetic cardiomyopathy (Figure 1).

### 3.5. Interpretation

The images in panels D–F underscore the pathological changes in diabetic cardiomyopathy, including hypertrophy and increased TGF-β1 immunoexpression, indicative of fibrotic processes and structural abnormalities (Figure 1).

Panels G–I demonstrate the mitigating effects of Vinpocetine on cardiac morphology and TGF-β1 immunoexpression in diabetic rats. The reduction in cardiomyocyte thickness and TGF-β1 immunoexpression suggests a potential protective role of Vinpocetine against fibrotic changes associated with diabetic cardiomyopathy (Figure 1).

These histopathological findings complement the quantitative data presented in Table 1, reinforcing the notion that Vinpocetine treatment attenuates structural abnormalities and fibrotic processes in the diabetic heart. The microscopic evidence supports the hypothesis that Vinpocetine exerts a protective and remodeling effect on cardiac tissue in the context of diabetic cardiomyopathy.

## 4. Discussion

The effects of vinpocetine administration on cardiac injury are still a relatively new topic in the scientific literature. The purpose of this study was to investigate the effects of Vinpocetine on diabetic cardiomyopathy. 

Previous studies have demonstrated that Vinpocetine, when administered at the exact dosage, effectively reduces inflammation of the lungs [20,21], injury-induced vasculature intimal hyperplasia [22], and atherosclerosis [23] in mouse models. In this study, Vinpocetine affected cardiac inflammatory and oxidant markers parallel to its effect on lung and vascular structures in rats. We can discuss how Vinpocetine exerts this effect by reducing oxidative stress and its vasodilator effects in rats. 

Randomized animal studies of type 1 and 2 diabetes have shown decreased diastolic and systolic contractile function, reduced cardiomyocyte contraction, and alterations in specific cardiomyocyte proteins [24,25]. Consequently, it can be inferred that the definition of DCM should encompass abnormal contractile function in cardiomyocytes affected by diabetes. A study by Zhou et al. found that NRG-1 enhances cardiac function and safeguards cardiomyocytes against sepsis-induced cardiomyopathy by inhibiting immune inflammation and excessive activation of the renin–angiotensin–aldosterone system [26]. On the other hand, in a study by Rochais et al., they observe that Neuregulin-1 promptly regulates the production of nitric oxide and the management of calcium in rat cardiomyocytes, serving as a vital factor in the development of the heart and the preservation of its functionality in adult individuals [27]. Moreover, in a study by Gupte et al., treatment with recombinant NRG-1 shows promise as a therapy for heart failure following a heart attack in patients with type 1 diabetes. It achieves this by decreasing the occurrence of myocardial fibrosis and apoptosis, as well as reducing the activity of enzymes that produce oxidants [28]. In this study, NRG-1 augments with the admission of Vinpocetine. From this result, Vinpocetine stops the degradation process of cardiomyocytes and improves it by regulating NO and calcium equilibrium. 

HIF-1 alpha is necessary for generating mitochondrial ROS and protecting the myocardium against injury caused by ischemia-reperfusion in mice during ischemic preconditioning [29]. On the other hand, in a study conducted by Xiaoou Wang, it was found that HIF-1 is crucial in the process of programmed cell death in heart muscle cells caused by the lack of oxygen and subsequent reoxygenation. By suppressing the activity of HIF-1, it is possible to decrease the damage to the heart muscle following a heart attack [30]. Moreover, M. Refaie et al. describe that Vinpocetine exerts cardioprotective effects against doxorubicin-induced damage through the modulation of HIF/VEGF and cGMP/cAMP/SIRT signaling pathways, alongside its anti-apoptotic, anti-inflammatory properties, and antioxidant properties [31]. In this study, HIF-1 diminishes in DCM rats and augments with the admission of Vinpocetine. As in MDA levels, HIF-1 levels also show the protective effect of Vinpocetine against oxidative stress. 

Troponin is the most used biomarker in practice for detecting myocardial damage [32,33] and is a direct reflection of myocardial hypoxia in plasma [34,35]. Pro-BNP is a prognostic and direct indicator of heart failure, especially in decompensated heart failure [36,37]. cTnT and pro-BNP are released from cardiomyocytes in proportion to the magnitude and extent of cardiac tissue damage caused by diabetic conditions. As the damage levels observed from cTnT and pro-BNP decrease in the Vinpocetine + Diabetic group, we can comment that this effect can be from the antioxidant anti-inflammatory effect as well as the vasodilator effect of Vinpocetine because vasodilatation can extend the diffusion time of oxygen and metabolites from blood to the myocardium and protect from catastrophic ischemia. 

In a similar study conducted by Mei-ping Wu et al. in mice, they described that Vinpocetine’s first target is Phosphodiesterase inhibitor 1 (PDEI) [38]. The PDE1 family is involved in various processes, such as controlling the growth of smooth muscle, facilitating learning and memory, promoting cardiac hypertrophy, and influencing olfaction [39,40,41]. In a study conducted by Vigneshwar, they describe that inhibition of PDE1 protects primary mouse cardiomyocytes from doxorubicin-induced cardiotoxicity by enhancing cell survival and inducing an inotropic response [42]. Moreover, in a study by Hana et al., they describe that DCM encompasses a range of cardiac abnormalities. The objective of this study is to examine the potential correlation between DCM and alterations in cyclic adenosine 3′-5′ monophosphate (cAMP) signaling, specifically focusing on cyclic nucleotide PDEs and cardiac PDEs exhibiting distinct and time-dependent control, which could assist in the advancement of novel treatment strategies for diabetic cardiomyopathy induced by type 1 diabetes [43]. On the other hand, Hanna, in the same study on PDE1 inhibitors, showed that cardiac PDEs show differential and time-specific regulation, potentially aiding in the development of new therapeutic approaches for type 1 diabetes-induced diabetic cardiomyopathy [43]. From this knowledge, we can speculate that the inhibitory effect of Vinpocetine on heart remodeling may be achieved by suppressing PDE1 activity. 

Similarly, genetic depletion of PDE1 attenuated cardiomyocyte hypertrophy, death, and fibrosis in vitro and in vivo [44]. A review conducted by Rosansarz informs that Angiotensin II (AT-II) and TGF-beta 1 collaborate in a signaling network to facilitate cardiac remodeling, a vital process in developing heart disease and clinical outcomes [45]. Similar to the results of Mei-ping Wu [35] in this study, we demonstrate that Vinpocetine mediates the fibrotic process of the heart by reducing TGF-1beta after cardiac injury, which occurs in diabetes. In a study conducted by Connely et al., they describe that diabetic cardiomyopathy develops in rats due to an increased tissue renin–angiotensin system and hyperglycemia. This condition is characterized by fibrosis and myocyte hypertrophy, which may be caused by the activation of transforming growth factor beta due to diabetes [46]. This is more proof that the Vinpocetine healing effect on cardiac muscle thickness was reduced after Vinpocetine admission. In this study, the plasma glucose levels did not change with Vinpocetine, so this clarifies that the effect of Vinpocetine does not depend on glucose–insulin regulation. 

Excessive levels of insulin and glucose can cause the activation of the TGF-β1 pathway and disrupt the breakdown of the extracellular matrix (ECM) [47]. Overexpression of TGF-beta 1 in the heart can stimulate nonadaptive cardiac remodeling and myocardial fibrosis, but these effects can be prevented by antagonism [38,48].

Fein et al. demonstrated that the hypertensive diabetic rats had increased cardiac necrosis and fibrosis, with a greater degree of right ventricular damage, which may ultimately result in congestive heart failure or arrhythmia [49]. On the other hand, Litwin et al. described that rats with diabetes display early signs of dilated cardiomyopathy, a condition that can be reversed by administering insulin [50]. In this study, with diabetic conditions, left and right ventricle thickness is increased after diabetic decrease with Vinpocetine admission. This healing effect occurs thanks to the suppression of TGF-beta 1 in tissue and in plasma by Vinpocetine.

Histological results in studies [38,51,52] show that Vinpocetine efficiently mitigates pathologic cardiac remodeling by inhibiting the hypertrophic growth of cardiac myocytes, the activation of fibroblasts, and the expression of genes associated with fibrosis. In this study, apart from the similarity of these results, unlike the other study, TGF-beta 1 expression was also histologically examined in the heart tissue, and the decrease in TGF-beta 1 after Vinpocetine admission shows that Vinpocetine prevents the healing of the heart muscle with fibrosis and reveals a myocyte protective effect.

## 5. Conclusions

In this study, we describe the protective effect of Vinpocetine with its vasodilatory, anti-inflammatory, and antioxidant effects, and probably via the inhibition of TGF-Beta 1, to protect the heart from maladaptive processes after a cardiac injury like diabetic cardiomyopathy. To summarize, Vinpocetine treatment exhibited notable protective effects against diabetic cardiomyopathy, as indicated by enhancements in cardiac morphology, immunohistochemistry, and modulation of diverse biochemical markers. The results indicate that Vinpocetine may have a beneficial effect in reducing the negative impact of diabetes on heart function, potentially serving as a therapy.

## Figures and Tables

**Table 1:** Morphological analysis of the Normal, Diabetes + saline, and Diabetes + Vinpocetine groups. Data are expressed as mean ± SEM. There is no significant difference between groups containing the same letter (Multiple Comparison Test Corrected Bonferroni was used).

	Normal Control (Group-1)	Diabetes and Saline Treatment (Group-2)	Diabetes and Vinpocetine (5 mg/kg) Treatment (Group-3)	*p*
Cardiac muscle cell thickness (% of control)	100 ^a^	119.2 ± 2.3 ^b^	108.5 ± 1.1 ^c^	<0.01
Left ventricle Cardiac muscle cell thickness (µm)	28.1 ± 0.5 ^a^	33.7 ± 1.6 ^b^	29.8 ± 0.7 ^a^	<0.01
Left ventricle thickness (mm)	3.12 ± 0.3 ^a^	3.85 ± 0.2 ^b^	3.41 ± 0.09 ^c^	<0.01
Right ventricle thickness (mm)	0.95 ± 0.08 ^a^	1.16 ± 0.1 ^b^	1.04 ± 0.06 ^a^	<0.01
Interventricular septum thickness (mm)	2.23 ± 0.1 ^a^	2.69 ± 0.2 ^b^	2.25 ± 0.3 ^a^	<0.01

**Table 2:** Biochemical analysis of the Normal, Diabetes + saline, and Diabetes + Vinpocetine groups. Data are expressed as mean ± SEM. There is no significant difference between groups containing the same letter (Multiple Comparison Test Corrected Bonferroni was used). TGF-β: Transforming growth factor-β, MDA: malondialdehyde, HIF: hypoxia-induced factor.

	Normal Control (Group-1)	Diabetes and Saline Treatment (Group-2)	Diabetes and Vinpocetine (5 mg/kg) Treatment (Group-3)	*p*
Immunoexpression TGF-β1 percent (%)	1.2 ± 0.3 ^a^	15.7 ± 2.8 ^b^	4.9 ± 1.6 ^a^	<0.001
Blood glucose (mg/dL)	75.4 ± 2.3 ^a^	420.8 ± 6.9 ^b^	401.6 ± 8.9 ^b^	<0.01
Plasma TGF-Beta (ng/mL)	17.4 ± 1.8 ^a^	72.5 ± 4.4 ^b^	39.6 ± 2.1 ^c^	<0.001
Plasma MDA (nM)	73.6 ± 5.2 ^a^	349.1 ± 7.6 ^b^	158.1 ± 6.5 ^c^	<0.001
Cardiac HIF-1alpha (pg/mg)	7.3 ± 0.1 ^a^	2.8 ± 0.3 ^b^	4.2 ± 0.1 ^c^	<0.01
Cardiac neuregulin-1β (pg/mg)	32.8 ± 1.7 ^a^	20.6 ± 0.8 ^b^	25.3 ± 2.2 ^b^	<0.01
Plasma Pro-BNP (pg/mL)	3.6 ± 0.8 ^a^	9.8 ± 0.5 ^b^	6.06 ± 0.3 ^b^	<0.01
Plasma Troponin T (pg/mL)	0.72 ± 0.2 ^a^	2.1 ± 0.09 ^b^	1.5 ± 0.1 ^b^	<0.01

## Data Availability

All the data for this study are presented in the published article. Any further details are available from the corresponding author (Ejder Saylav Bora, saylavbora@hotmail.com) upon a reasonable request.
